# Enhancement of growth and Cannabinoids content of hemp (*Cannabis sativa*) using arbuscular mycorrhizal fungi

**DOI:** 10.3389/fpls.2022.845794

**Published:** 2022-07-26

**Authors:** Wasan Seemakram, Jakkapat Paluka, Thanapat Suebrasri, Chanon Lapjit, Somdej Kanokmedhakul, Thomas W. Kuyper, Jindarat Ekprasert, Sophon Boonlue

**Affiliations:** ^1^Department of Microbiology, Faculty of Science, Khon Kaen University, Khon Kaen, Thailand; ^2^Natural Products Research Unit, Department of Chemistry and Center of Excellence for Innovation in Chemistry, Faculty of Science, Khon Kaen University, Khon Kaen, Thailand; ^3^Department of Medical Science, Faculty of Allied Health Sciences, Nakhon Ratchasima, Thailand; ^4^Department of Horticulture, Faculty of Agriculture, Khon Kaen University, Khon Kaen, Thailand; ^5^Cannabis Research Institute, Khon Kaen University, Khon Kaen, Thailand; ^6^Soil Biology Group, Wageningen University and Research, Wageningen, Netherlands

**Keywords:** *Rhizophagus aggregatus*, *Rhizophagus prolifer*, plant growth promotion, organic agriculture, *Cannabis sativa* KKU05

## Abstract

This study aimed to investigate the efficiency of arbuscular mycorrhizal fungi (AMF) to promote growth and cannabinoid yield of *Cannabis sativa* KKU05. A completely randomized design (CRD) was conducted with six replications for 60 days. Two different species of AMF, *Rhizophagus prolifer* PC2-2 and *R. aggregatus* BM-3 g3 were selected as inocula and compared with two non-mycorrhizal controls, one without synthetic fertilizer and one with synthetic NPK fertilizer. The unfertilized non-mycorrhizal plants had the lowest performance, whereas plants inoculated with *R. aggregatus* BM-3 g3 performed best, both in terms of plant biomass and concentrations of CBD and THC. There were no significant differences in plant biomass and cannabinoid concentrations between non-mycorrhizal plants that received synthetic fertilizer and mycorrhizal plants with inoculum of *R. prolifer* PC2-2. Our data demonstrate the great potential for cannabis cultivation without risking deterioration of soil structure, such as soil hardening and increased acidity, which might be induced by long-term use of synthetic fertilizer.

## Introduction

Cannabis (*Cannabis sativa*) is an annual plant that is widely cultivated because of its industrial and medicinal importance (Preedy, 2017). Cannabis plants and their extracts offer several applications such as fibers, building materials, confectionery and beverages, dietary supplements, cosmetics, medicinal drugs, and substances for recreational purposes (Fike, 2016; Schluttenhofer and Yuang, 2017; Crini et al., 2020; Wimalasiri et al., 2021; Xu et al., 2021). In Thailand, the cultivation of cannabis has become legal since 2020 when the Ministry of Public Health announced that cannabis plants and extracts are no longer classified as a Category 5 narcotic in Thailand’s Narcotics Act. Consequently, its cultivation has gained increasing interest. Cannabis accumulates cannabinoids as secondary metabolites, which are mainly in the form of Δ9-tetrahydrocannabinol (THC), cannabinol (CBN) and cannabidiol (CBD; Barrales-Cureño et al., 2020). These cannabinoids are produced and stored in the trichomes or hair glands that are abundant around the female flower. Apart from cannabinoids, a considerable amount of terpenoid-like and phenolic compounds, which can be used as medicine, are found as metabolites (Flores-Sanchez and Verpoorte, 2008; Matteucci et al., 2016). The US Food and Drug Administration (FDA) has approved the application of cannabinoids for medicinal purposes, for example, the use of THC-based medication for nausea in patients undergoing cancer chemotherapy and for stimulating appetite in patients with AIDS weight loss syndrome. Furthermore, CBD has been used for the treatment of severe childhood epilepsy such as Dravet syndrome and Lennox–Gastaut syndrome (Nora and Volkow, 2020). Accordingly, research how to improve plant growth and increase cannabinoids content has gained more attention. The most common and easily accessible practice is the use of synthetic fertilizer to enhance plant production. However, lower soil quality, notably a reduction of beneficial soil microbes, and undesirable effects of overuse of synthetic fertilizer and toxic chemical residues have been observed after the long-term use of synthetic fertilizers (Chandini et al., 2019). In order to reduce the use of synthetic fertilizers to promote the growth and yield of cannabis, plant growth promoting microorganisms (PGPM) are of interest and they have been extensively studied due to their environmentally-friendly nature (Andre et al., 2016; Pagnani et al., 2018).

Arbuscular mycorrhizal fungi (AMF) are a group of fungi belonging to the subphylum Glomeromycotina, phylum Mucoromycota (Spatafora et al., 2016) that live in a mutualistic symbiosis with plant roots of the majority (>80%) of terrestrial plant species (Boonlue et al., 2012; Seemakram et al., 2021). AMF develop extraradical mycelia that extend the depletion zone that develops around roots, and facilitate the acquisition of nutrients of low mobility. Furthermore, AMF can help plants to adjust the osmotic balance within the cells (Rahimzadeh and Pirzad, 2017; Nacoon et al., 2021). AMF can also help the plants to grow under unfavorable environmental conditions, such as arid conditions, by increasing water uptake (Meddich et al., 2015; Begum et al., 2019). They also improve plant tolerance to a variety of stresses including salinity, herbivory, plant diseases, and heavy-metal pollution (Ahanger et al., 2014; Salam et al., 2017). Due to these beneficial features, AMF are a good candidate for acquiring more nutrients and water for plants under conditions where those resources are limited, and hence likely increase plant biomass and the production of these cannabinoids (Ahmed and Hijri, 2021). Beneficial effects of AMF on performance of *C. sativa* were reported by Citterio et al. (2005; enhanced performance in heavy-metal polluted soil) and Kakabouki et al. (2021a; biomass and nitrogen and phosphorus content). There is still no report on the use of AMF for cultivation to promote growth of cannabis and increase cannabinoid yield.

In this study, two different species of AMF, *Rhizophagus aggregatus* (N.G. Schenck & G.M. Smith) C. Walker BM-3 g3 and *Rhizophagus prolifer* (Dalpé & Declerck) C. Walker & Schluessler PC2-2 were investigated in a pot trial for their efficiency to promote growth and yield of cannabinoids. Both AMF species promoted growth in the legume family [Siamese rosewood (*Dalbergia cochinchinensis*) and Burma padauk (*Pterocarpus macrocarpus*)] and the production of plant secondary metabolites in Jerusalem artichoke (*Helianthus tuberosus*) plants (Nacoon et al., 2021; Seemakram et al., 2021). However, there is still no report investigating the effects of both AMF species on the growth and production of secondary metabolites of cannabis. The results obtained from this work could probably pave ways to an *in-situ* application of AMF for the sake of increasing cannabis cultivation in Thailand.

## Materials and methods

### Preparation of cannabis plants

*Cannabis sativa* KKU05 (Hemp) cuttings were provided by the Cannabis Research Institute, Khon Kaen University, Khon Kaen, Thailand. Cuttings were cloned from 8-week mother plants, which were at a vegetative stage. Branches with a couple of nodes below the top were cut off. These cuttings were transferred to trays containing sterilized peat moss and subsequently grown under a plastic dome in order to lower levels of transpiration and to keep the cuttings well hydrated. Cuttings were grown for 2 weeks at 28°C, with 16-h light per day using a RGB (a mixture of red, blue and green light) LED light having an intensity of 120 μmol m^−2^ s^−1^ before the mycorrhizal inoculum was added.

### AMF preparation

The AMF strains used in this study were *R. prolifer* PC2-2 and *R. aggregatus* BM-3 g3, obtained from the Mycorrhizal and Fungal Technology Laboratory, Department of Microbiology, Faculty of Science, Khon Kaen University, Thailand. AMF spore propagation was carried out by a pot culture technique using maize (*Zea mays*) as host plant as described by Boonlue et al. (2012). Briefly, soil was sterilized twice before filling 20-cm diameter plastic pots. Maize seeds were surface-sterilized by soaking in a 10% sodium hypochlorite solution for 30 min, then added to the soil. Maize was grown in a greenhouse at 30–35°C with daily irrigation using tap water. After 90 days, irrigation was stopped in order to allow the plants to dry for 5 days, by which spore formation of AMF was induced (Seemakram et al., 2021). Plants were subsequently cut off just above the soil surface. Finally, dried soil was crushed into finely ground particles. The purity of AMF spores and the total number of spores were determined using the sucrose centrifugation method (Daniels and Skipper, 1982). Dried soils containing AMF spores, mycelial fragments, and colonized roots were then used as soil inoculum.

### Soil preparation

The sandy loam soil used in this study was the same soil as used by Nacoon et al. (2020). It had the following properties: pH 5.27, 0.14 dS m^−1^ electrical conductivity (EC), 3.9 g soil organic matter kg^−1^, 160 mg total N kg^−1^ (C:N ≈ 12), 5 mg extractable (P-Bray) P kg^−1^, 36 mg exchangeable K kg^−1^, 125 mg exchangeable Ca kg^−1^ and 56 mg exchangeable Na kg^−1^. Prior to the experiment, rocks, wood chips and plant debris were removed. Soil samples were sterilized by autoclaving at 121°C, 15 psi for 120 min. The soils were left to cool down at room temperature overnight, before autoclaving a second time using the condition as previously described. Soils were then filled into 30-cm diameter pots with a total soil volume of 7.5 l per pot for cultivation of cannabis cuttings.

### Experimental design

The experiment was conducted in an enclosed greenhouse at the Cannabis Research Institute, Khon Kaen University, Khon Kaen, Thailand. The experiment was a completely randomized design (CRD), consisting of 4 treatments with 6 replications, for a total of 24 pots. Four treatments were as follows: T1: control plant without microbial inoculum; T2: plants with added synthetic fertilizer, but without microbial inoculum; T3: plants inoculated with *R. prolifer* PC2-2; and T4: plants inoculated with *R. aggregatus* BM-3 g3. Treatment T2 with synthetic fertilizer was set up by adding N, P and K fertilizer of 15–15-15 into the soil at a ratio of 250 kg ha^−1^ as suggested by Deng et al. (2019). The treatment with AMF was conducted by inoculating AMF spores at a position adjacent to plant roots at a concentration of 200 spores pot^−1^ on the first day of planting. The pots were then covered with soil and watered with filtered tap water. Plant samples were collected for physico-chemical analysis 2 months after plants were transferred to pots.

### Plant performance

Plant growth parameters including plant height, trunk diameter, plant dry weight, total number of branches and florets, leaf area, and SPAD were measured at the harvest stage, 2 months after the plants were transferred to pots. The height of cannabis plants was measured by a standard stick method. Diameter of cannabis stem was measured at 2.5 cm above the ground using a Vernier caliper (Mitutoyo, Japan). To determine biomass, plants were dried in an oven at 80°C until a constant weight was obtained. The total number of florets per pot was counted (Note that the number of florets refers to the total number of flowers). All fresh leaves were collected for measuring leaf area using a LI-3100C area meter (LI-COR Bioscience, Thailand). The greenness of leaves was determined from the second expanded leaves from the top of the main stem using the SPAD meter (SPAD 250 + KONICA MINOLTA, Japan).

Roots traits were determined on three plants, randomly chosen for each treatment. Fresh roots, at an amount of 10% of the total number of roots in each treatment, were randomly collected. Root samples were scanned using a root scanner (Epson perfection V700 photo) in order to determine root length, root diameter, and root surface area. Dry weight of the scanned samples was determined and specific root length calculated. Root tissue density was also calculated, assuming roots as cylinders. Data were analyzed using the WINRHIZO Pro2004a software (REGENT Instruments Inc., QC, Canada).

Of the three other plants, dried stem, leaves, and root were ground into powder for analysis of nitrogen. N was measured by the micro-Kjeldahl method (Jackson, 1967) followed by the indophenol blue method (Schuman et al., 1973).

### Mycorrhizal root colonization

Roots were randomly collected to assess mycorrhizal root colonization intensity. Roots were cleared with 10% KOH for 3 min at 95°C, and then acidified in 2% HCl overnight. Root samples were stained with 0.05% trypan blue solution (Koske and Gemma, 1989) before cutting into 1-cm long pieces. Fractional mycorrhizal colonization was determined according to the method described by Trouvelot et al. (1986). Cellular structures of AMF including vesicles, arbuscules, and hyphae were observed using a microscope at magnification of 40× (SMZ745T Nikon, Japan).

### Extraction and quantification of cannabinoids

Three dried floret samples per treatment (around 5 g each) were ground to powder and heated at 100°C for 3 h before the extraction of cannabinoids. Hexane was mixed with the sample at a ratio of hexane (ml):sample (g; 10,1 v/w) and then kept at 4°C for 24 h. The solvent fraction was retrieved and then filtered using a vacuum funnel (Buchner funnel, Fisher Scientific, Sweden). Solvent was evaporated using a rotary evaporator (RE300/MS Rotary Evaporator, TEquipment, United States) to obtain crude extract of cannabinoids. The presence of cannabinoids in the crude extract samples was determined by high-performance liquid (HPLC) chromatography analysis (Agilent, Germany).

Types of cannabinoid compounds were determined by comparing retention times to the chromatograms of the standard compounds which were Cannabidiol (CBD) Tetrahydrocannabinol (THC), Cannabinol (CBN), Cannabidiolic acid (CBDA), Tetrahydrocannabinolic acid (THCA) and Cannabigerol (CBG). Quantification of cannabinoid metabolites was carried out by extrapolating peak areas of the target compounds with the calibration curves of the corresponding compounds (Zivovinovic et al., 2018). The mobile phase was acetonitrile with 0.1% triethylamine (TEA) diluted in distilled water at a ratio of 40:60 v/v, and the pH was adjusted to 2.2. Prior to use, the mobile phase solution was filtered through a hydrophilic PTFE membrane filter (0.5 μm, ADVANTEC, Toyo Roshi Kaisha, Ltd. Tokyo, Japan). The HPLC condition was as follows: the flow rate was 1.5 ml min^−1^; column temperature 35°C; monitoring wavelength 228 nm using a UV absorbance detector; column, CORTECS Shield RP18 (2.7 μm particle size, 4.6 mm × 150 mm).

### Statistical analysis

All data were analyzed using Statistix 10 software. Data were tested for normality (Shapiro-Wilks) and homogeneity of variance (Bartlett’s test). One-way analysis of variance (ANOVA) was performed to analyze differences among the means of the data. Fisher’s Least Significant Difference (LSD) was applied to analyze significant differences between treatments at a 95% confidence interval (*p* ≤ 0.05).

## Results

### Aboveground plant performance

**Figure 1:**
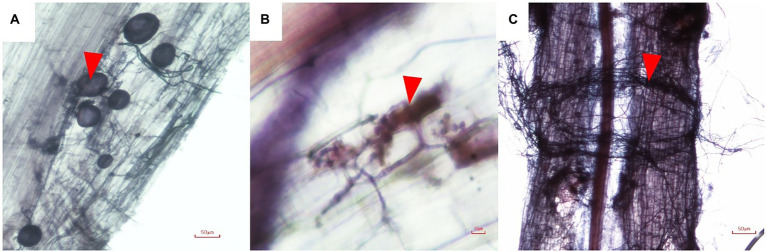
Morphology of AMF in root of *Cannabis sativa* **(A)**, vesicles (arrow head); **(B)**, Arum-type arbuscules (arrow head), and **(C)** hyphae around plant roots (arrow head).

At 60 DAT, mycorrhizal plants outperformed non-mycorrhizal plants in stem height, stem diameter, stem dry weight, leaf area, leaf weight, SPAD index, number of branches, and number and weight of florets, however the differences were usually not significant when plants were inoculated with *R. prolifer* PC2-2, while the differences in performance by plants inoculated with *R. aggregatus* BM-3 g3 were always significant. Plants that were fertilized with synthetic fertilizer performed somewhat better than non-fertilized plants, however differences were also often not significant. Plants inoculated with *R. prolifer* PC2-2 did perform equally well as fertilized plants for most performance parameters, while plants inoculated with *R. aggregatus* BM-3 g3 significantly outperformed plants that received synthetic fertilizer (Table 1). Plant morphology showed more branching in plants inoculated with mycorrhizal fungi than in plants that were non-inoculated (T3 and T4; Table 1), irrespective of the level of fertilization (Figure 1). Nitrogen concentrations were highest in plants inoculated with *R. aggregatus* BM-3 g3, and the difference between this treatment and the non-fertilized plants was significant. There was no statistically significant difference in nitrogen concentrations between fertilized plants and (non-fertilized) mycorrhizal plants.

**Table 1:** Aboveground plant performance parameters of *Cannabis sativa* KKU05 grown under different conditions at 60 DAT.

Treatments	Height (cm)	Diameter (mm)	Leaf area (cm^2^)	SPAD	Total number of branches	Total number of florets	Leaf dry weight (g)	Stem dry weight (g)	Florets dry weight (g)	N concentration (mg g^−1^)
T1	43 ± 1.78 b	3.7 ± 0.36b	258.47 ± 10.33c	47.48 ± 2.13b	1.67 ± 0.12c	18.00 ± 2.00b	1.22 ± 0.36c	0.90 ± 0.19b	5.83 ± 1.55b	16.60 ± 0.11b
T2	58 ± 1.80b	4.9 ± 0.32ab	485.82 ± 11.08b	54.43 ± 0.83a	5.00 ± 0.24b	20.00 ± 2.64b	2.20 ± 0.70b	1.40 ± 0.24ab	8.80 ± 0.85ab	19.99 ± 0.32ab
T3	52 ± 1.52b	4.9 ± 0.02ab	435.02 ± 20.78bc	51.67 ± 1.07ab	7.00 ± 0.15b	26.00 ± 1.11b	1.80 ± 0.04bc	1.43 ± 0.11ab	9.30 ± 1.06ab	17.75 ± 0.05ab
T4	80 ± 1.50a	5.2 ± 0.80a	974.17 ± 28.54a	52.83 ± 1.09a	10.67 ± 0.27a	46.00 ± 1.65a	4.40 ± 0.90a	3.30 ± 0.60a	11.80 ± 1.74a	21.12 ± 0.21a
F-test	^**^	^*^	^**^	^*^	^**^	^**^	^**^	^**^	^*^	^*^

Numbers with different letters in each column indicate significant differences according to the LSD test. Each value in the graph represents the mean ± SD; (*n* = 6). T1: control without microbial inoculum, T2: Synthetic fertilizer, T3: Rhizophagus prolifer PC2-2 and T4: R. aggregatus BM-3 g3.

* indicates significant difference at *p* ≤ 0.05.

** indicates significant difference at *p*≤ 0.01.

### Root traits and mycorrhizal colonization

Root dry weight was largest for plants inoculated with *R. aggregatus* BM-3 g3, whereas there were no differences between the three other treatments. Total root length and surface area were also largest for plants inoculated with *R. aggregatus* BM-3 g3 and smallest for non-fertilized, non-mycorrhizal plants and plants inoculated with *R. prolifer* PC2-2. Root diameter, specific root length, and root tissue density did not show significant effects of treatment (Table 2).

**Table 2:** Belowground plant performance parameters of *Cannabis sativa* KKU05 grown under different conditions at 60 DAT.

Treatments	Length (m)	Surface area (cm^2^)	Root dry weight (g)	Average diameter (mm)	Specific root length (m g^−1^)	Root tissue density (g cm^−3^)
T1	98.23 ± 7.12b	689.46 ± 19.01b	0.60 ± 0.17b	0.23 ± 0.18a	187.20 ± 6.68a	0.10 ± 0.00a
T2	139.40 ± 3.12ab	967.85 ± 23.97ab	0.80 ± 0.05b	0.22 ± 0.46a	174.50 ± 7.76a	0.12 ± 0.00a
T3	99.60 ± 8.20b	692.63 ± 18.94b	0.80 ± 0.45b	0.21 ± 0.11a	154.60 ± 18.19a	0.14 ± 0.02a
T4	235.30 ± 9.10a	1730.58 ± 19.76a	2.21 ± 0.11a	0.23 ± 0.10a	107.00 ± 33.17a	0.14 ± 0.01a
F-test	^*^	^**^	^**^	n.s.	n.s.	n.s.

Numbers with different letters in each column indicate significant differences according to the LSD test. Each value in the graph represents the mean ± SD; (*n* = 6). T1: control without microbial inoculum, T2: Synthetic fertilizer, T3: Rhizophagus prolifer PC2-2 and T4: R. aggregatus BM-3 g3.

* indicates significant difference at *p*≤ 0.05;

** indicates significant difference at *p*≤ 0.01.

**Figure 2:**
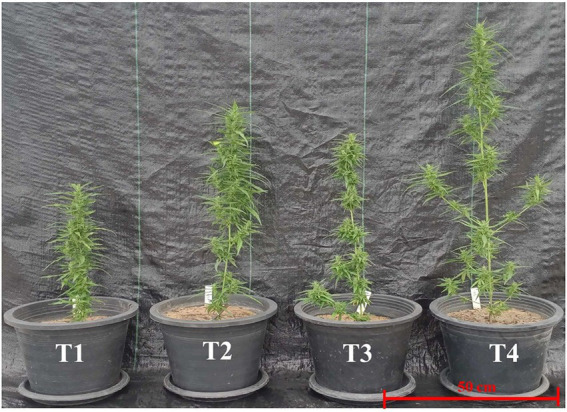
Plants of *Cannabis sativa* KKU05 grown under different conditions: T1, control without AMF inoculum; T2, Non-mycorrhizal plants with synthetic NPK fertilizer; T3, Plants inoculated with *Rhizophagus. prolifer* PC2–2 and T4, Plants inoculated with *R. aggregatus* BM-3 g3 (Photos were taken 60 days after the start of treatment).

Non-inoculated plants remained free of mycorrhizal root colonization, whereas inoculated plants showed the presence of hyphae, arbuscules and vesicles (Figure 2). Root colonization of plants inoculated with *R. aggregatus* BM-3 g3 was 21%, significantly higher than colonization of plants inoculated with *R. prolifer* PC2-2 (13%). Mycorrhizal fungal spores were observed in pots of both mycorrhizal treatments, while they were absent in pots of the treatments without mycorrhizal inoculum (data not shown).

### Cannabinoid concentrations

HPLC chromatograms showed that plants produced CBD, CBDA, CBG and THC regardless of the type of treatment. No THCA was found in any crude extract which was due to the decarboxylation of THCA into THC during drying floret samples by heating at 100°C (Taschwer and Schmid, 2015). Concentrations of CBD and THC were lowest in non-fertilized non-mycorrhizal plants and highest in plants inoculated with *R. aggregatus* BM-3 g3, while plants that received synthetic fertilizer and plants that were inoculated with *R. prolifer* PC2-2 had intermediate concentrations. There were no significant treatments effects on concentrations of CBDA and CBG (Table 3). Because of treatment effects on floret weight, non-mycorrhizal plants had the lowest content of all four psychoactive compounds, whereas those inoculated with *R. aggregatus* BM-3 g3 had the highest content. There were no significant differences in the content of psychoactive compounds between plants that received synthetic fertilizer and plants inoculated with *R. prolifer* PC2-2 (Table 3).

**Table 3:** Contents of major cannabinoids produced by *Cannabis sativa* KKU05 grown under different conditions at harvest stage (60 days).

Treatment	Concentration in dry sample (mg g^−1^)				Total of cannabinoids (g)			
	CBD	CBDA	CBG	THC	CBD	CBDA	CBG	THC
T1	24.56 ± 0.07c	0.40 ± 0.17a	2.00 ± 0.11a	1.20 ± 0.00b	0.15 ± 0.04c	0.003 ± 0.00b	0.011 ± 0.00b	0.007 ± 0.00c
T2	26.39 ± 1.07bc	1.04 ± 1.10a	1.62 ± 0.60a	1.20 ± 0.04b	0.20 ± 0.15bc	0.014 ± 0.01a	0.014 ± 0.01ab	0.010 ± 0.00b
T3	31.70 ± 0.75ab	0.90 ± 0.55a	1.53 ± 0.02a	1.50 ± 0.09ab	0.27 ± 0.01ab	0.013 ± 0.00ab	0.014 ± 0.00ab	0.013 ± 0.00b
T4	32.28 ± 0.23a	1.24 ± 0.66a	2.00 ± 0.01a	1.65 ± 0.02a	0.32 ± 0.01a	0.012 ± 0.00ab	0.021 ± 0.00a	0.016 ± 0.00a
F-test	^**^	n.s.	n.s.	^**^	^**^	^*^	^*^	^**^

Numbers with different letters in each column indicate significant differences according to the LSD test. Each value in the graph represents the mean ± SD; (*n* = 6). T1: control without microbial inoculum, T2: Synthetic fertilizer, T3: Rhizophagus prolifer PC2-2 and T4: R. aggregatus BM-3 g3.

* indicates significant difference at *p*≤ 0.05;

** indicates significant difference at *p*≤ 0.01.

## Discussion

According to our previous studies, both AMF species showed high efficiency in enhancing plant growth and biomass in two woody legumes (Siamese rosewood and Burma padauk; Seemakram et al., 2021) and plant secondary metabolites in Jerusalem artichoke (Nacoon et al., 2021). Therefore, inoculation of these species was expected to play an important role in improving growth and production of secondary metabolites in cannabis. We compared the efficiency of both AMF species, *viz. R. prolifer* PC2-2 and *R. aggregatus* BM-3 g3, on the promotion of growth and cannabinoid contents of *C. sativa* KKU05 with the effect of application of synthetic NPK fertilizer. Both inoculation with AMF and synthetic fertilizer increased performance of cannabis and increased yield of the cannabinoids compared to an unfertilized control. Inoculation with *R. prolifer* PC2-2 had an effect that was comparable to fertilizer application, whereas inoculation with *R. aggregatus* BM-3 g3 had significantly more beneficial effects than the application of synthetic fertilizer.

Both AMF strains colonized the roots of cannabis, confirming earlier reports that the species is mycorrhizal (Citterio et al., 2005; McPartland, 2018; Kakabouki et al., 2021a). *Rhizophagus aggregatus* BM-3 g3showed higher fractional colonization that *R. prolifer* PC2-2 (21% versus 13%). Higher fractional colonization of cannabis roots than in our study was reported in a study with *Rhizophagus irregularis*, *viz.* up to 30% (Kakabouki et al., 2021a), 42% with *Funneliformis mosseae* (Citterio et al., 2005), or 30–50% for three different species of AMF (Zalaghi et al., 2020). Our data are similar to mycorrhizal colonization of industrial hemp of 19% reported by Zielonka et al. (2021) in Poland but much higher than another study from that country where only colonization of 1% was reported (Zubek et al., 2012). Zielonka et al. (2021) reported as most common AMF species associated with industrial hemp various species of the genus *Funneliformis*, such as *F. mosseae*, *F. caledonium* and *F. geosporum*. Due to its forbidden nature of cannabis in the past, scientific knowledge on microbial diversity in cannabis cultivation, and especially that of variants for recreational or medicinal purposes, is limited (Ahmed and Hijri, 2021). To the best of our knowledge our study is the first to demonstrate effects of the mycorrhizal symbiosis on the production of cannabinoids.

Since most cannabinoid compounds accumulate within the buds and flowers of cannabis (Potter, 2014), an increase in both the quality and quantity of florets is considered crucial for judging the quality of plant growth promoting substances. Plants treated with our AMF inoculum produced a higher number of florets and floret dry mass than the untreated plants, both when fertilized and when non-fertilized. Nutrient concentrations in cannabis have a large effect on the amount of cannabinoid compounds. Gorelick and Bernstein (2017) reported that an increased mineral-nutrient concentrations such as Ca, Mg, N and K led to an increase in CBD and THC concentrations, while Caplan et al. (2017) noted beneficial effects of an organic amendment on THC concentrations. Our results support that finding as plants that were fertilized with NPK outperformed unfertilized control plants in the amount of biomass produced, while at the same time exhibiting slightly higher N concentrations. It has been observed that the production of cannabinoids is limited by phosphorus (Cockson et al., 2020) and that almost all of P acquired is found in inflorescences (Shiponi and Bernstein, 2021). These latter authors also observed a negative correlation between floret yield and cannabinoid concentration and suggested, in line with Imo (2012), that dilution was responsible for that negative concentration. However, in our case the plants with the highest floret number and mass also exhibited the highest concentrations of the cannabinoids CBD and THC, suggesting that dilution does not always occur. Because of the crucial role of phosphorus nutrition in cannabinoid concentrations, it is not surprising that inoculation with AMF could mimic the effect of a fertilizer application. However, whereas inoculation with *R. prolifer* PC2-2 resulted in cannabis plants of the same size and cannabinoid concentration as fertilized plants, inoculation with *R. aggregatus* BM-3 g3 resulted in larger plants with a large floret biomass, and additionally significantly higher concentrations of cannabinoids. Avio et al. (2018) listed a number of plant species from different families where inoculation with AMF both increased plant biomass and concentrations of bio-active compounds. However, none of the studies listed about mycorrhizal effects in enhancing concentrations of secondary, bio-active compounds has included the effects of synthetic fertilizers for comparative purposes. Our work is then the first to show that the effects of AMF to enhance production of bio-active compounds can exceed the effects of synthetic fertilizer.

Avio et al. (2018) suggested a mechanistic explanation for differential synthesis of such bio-active compounds in mycorrhizal and non-mycorrhizal plants in the link between the expression of these compounds and the mycorrhizal role in priming plant defense responses against pathogens. More specifically they suggested a major role for plant hormones such as jasmonic acid and abscisic acid. Next to these hormones a further role for strigolactones could be suggested, as strigolactones could modulate shoot branching. Plants inoculated with *R. aggregatus* BM-3 g3 showed enhanced shoot branching (Figure 1). Moreover, the effects of AMF to enhance plant quality during the flowering stage were also found in other flowering plants (Asrar et al., 2012; Gholamhoseini et al., 2013). Further research in the separate roles of nutrients (phosphorus) and plant hormones in the interplay between plant roots and mycorrhizal fungi (Kuyper et al., 2021) is recommended. It would also be relevant to test a larger number of species of AMF in order to predict which fungal species contribute more to cannabinoid yield. The strain of *R. aggregatus* BM-3 g3, which had the largest beneficial effect, on plant biomass and cannabinoid content and yield was also very beneficial in enhancing drought tolerance of Jerusalem artichoke (Nacoon et al., 2021). However, it is unlikely that the ability of a fungal species to enhance plant performance to a larger extent than other species of AMF is a general attribute of that fungal species. While many of the examples of a mycorrhizal effect on yield and concentrations of bio-active compounds that are listed in Avio et al. (2018) involve species of the genera *Rhizophagus* and *Funneliformis*, this listing might as much reflect standard research practices, where these species are most commonly used in experiments, rather than intrinsic fungal species-specific effects. *Rhizophagus* spp. play an important role in promoting growth of many types of plants due to their ability to increase plant photosynthesis (Djighaly et al., 2018; Sarr et al., 2020; Kakabouki et al., 2021a). The use of *Rhizophagus* inoculum to enhance cannabinoid yield in cannabis plants has never been mentioned before.

Not only AMF can have a beneficial effect on cannabinoid concentrations. Kakabouki et al. (2021b) found that colonization by *Trichoderma harzianum* increased CBD concentration in two varieties of *C. sativa*, however there was no significant increase in CBD yield per plant. Inoculation with *T. harzianum* also increased fractional root colonization, and their data no not allow disentangling effects of the ascomycete from the glomeromycotan fungus. Furthermore, *T. harzianum* increased CBD concentration only with ~12% compared to the control, much less than the effect of *R. aggregatus* BM-3 g3 (33% increase in CBD concentration, ~120% increase in CBD content). Plant growth-promoting rhizobacteria have also been suggested to enhance yield of bio-active substances. Pagnani et al. (2018) demonstrated that inoculation with a mixture of four plant growth-promoting rhizobacteria increased plant biomass and concentrations of CBD and THC compared to a control, and that the beneficial effect was similar to a N fertilizer application of 80 kg N ha^−1^. Comeau et al. (2021) investigated the effects of inoculation of individual and consortia of *Pseudomonas* and *Bacillus* species and observed no effects of inocula of single bacterial species but synergistic effects of species mixtures of around 30% in biomass enhancement. No data were provided on effects on cannabinoid concentrations, Species of *Bacillus* and *Pseudomonas*, inoculated singly or in consortia on roots of cannabis, did not have any protective effect against grey mold caused by *Botrytis cinerea*, whereas there was some positive effect on plant biomass (Balthazar et al., 2020). Lyu et al. (2019) suggested the beneficial use of those microbes, and especially those of the genera *Pseudomonas* and *Bacillus* for disease control and potentially for enhanced production of cannabinoids, but did not provide data to demonstrate these effects. Taghinasab and Jabaji (2020) and Vujanovic et al. (2020) also referred to the potential of the microbiome, while noting that there are very few empirical data to back up this suggestion.

Our study demonstrates the relationship between the growth and cannabinoid production of *C. sativa* and the presence of AMF. We found that inoculation with certain species of AMF promotes growth of hemp and increases cannabinoid contents more than the use of synthetic fertilizer, an effect that has not yet been previously reported. This finding thus paves the way for further development of AMF inoculum for industrial production of cannabis in a much greener manner than the conventional practice in which synthetic fertilizer is heavily used.

## Data availability statement

Publicly available datasets were analyzed in this study. This data can be found at: https://doi.org/10.1016/j.rhisph.2021.100363 and https://doi.org/10.1016/j.rhisph.2021.100308.

## Author contributions

WS and SB: conceptualization. CL and SK: resources. WS: writing—original draft preparation. WS, JP, TS, CL, SK, JE, TK, and SB: review and editing. All authors contributed to the article and approved the submitted version.

## Funding

This research was funded by the Industrial Postdoctoral Development for Agriculture, Food, Energy and Bio-materials for Future from Khon Kaen University, Thailand [grant no. (KKU-PMU-B) 63-027]. This work was partly supported by Research and Graduate Studies, Khon Kaen University, Thailand (grant no. RP64-3-Hemp Marijuana/001).

## Conflict of interest

The authors declare that the research was conducted in the absence of any commercial or financial relationships that could be construed as a potential conflict of interest.

## Publisher’s note

All claims expressed in this article are solely those of the authors and do not necessarily represent those of their affiliated organizations, or those of the publisher, the editors and the reviewers. Any product that may be evaluated in this article, or claim that may be made by its manufacturer, is not guaranteed or endorsed by the publisher.
